# Antibiotics for mastitis in breastfeeding women

**DOI:** 10.1590/1516-3180.20161343T1

**Published:** 2015-04-14

**Authors:** Shayesteh Jahanfar, Chirk Jenn Ng, Cheong Lieng Teng

## Abstract

**BACKGROUND:**

Mastitis can be caused by ineffective positioning of the baby at the breast or restricted feeding. Infective mastitis is commonly caused by *Staphylococcus aureus* . The prevalence of mastitis in breastfeeding women may reach 33%. Effective milk removal, pain medication and antibiotic therapy have been the mainstays of treatment.

**OBJECTIVES:**

This review aims to examine the effectiveness of antibiotic therapies in relieving symptoms for breastfeeding women with mastitis with or without laboratory investigation.

**METHODS:**

**Search methods:**

We searched the Cochrane Pregnancy and Childbirth Group's Trials Register (30 September 2012), contacted investigators and other content experts known to us for unpublished trials and scanned the reference lists of retrieved articles.

**Selection criteria:**

We selected randomised controlled trials (RCTs) and quasi-RCTs comparing the effectiveness of various types of antibiotic therapies or antibiotic therapy versus alternative therapies for the treatment of mastitis.

**Data collection and analysis:**

Two review authors independently assessed trial quality and extracted data. When in dispute, we consulted a third author.

**MAIN RESULTS:**

Two trials met the inclusion criteria. One small trial (n = 25) compared amoxicillin with cephradine and found no significant difference between the two antibiotics in terms of symptom relief and abscess formation. Another, older study compared breast emptying alone as 'supportive therapy' versus antibiotic therapy plus supportive therapy, and no therapy. The findings of the latter study suggested faster clearance of symptoms for women using antibiotics, although the study design was problematic.

**AUTHORS CONCLUSIONS:**

There is insufficient evidence to confirm or refute the effectiveness of antibiotic therapy for the treatment of lactational mastitis. There is an urgent need to conduct high-quality, double-blinded RCTs to determine whether antibiotics should be used in this common postpartum condition.

The full text of this review is available free-of-charge from: http://onlinelibrary.wiley.com/doi/10.1002/14651858.CD005458.pub3/epdf
